# The Effect of Submaximal Exercise Followed by Short-Term Cold-Water Immersion on the Inflammatory State in Healthy Recreational Athletes: A Cross-Over Study

**DOI:** 10.3390/jcm10184239

**Published:** 2021-09-18

**Authors:** Marta Pawłowska, Celestyna Mila-Kierzenkowska, Tomasz Boraczyński, Michał Boraczyński, Karolina Szewczyk-Golec, Paweł Sutkowy, Roland Wesołowski, Małgorzata Smoguła, Alina Woźniak

**Affiliations:** 1 Department of Medical Biology and Biochemistry, Ludwik Rydygier Collegium Medicum in Bydgoszcz, Nicolaus Copernicus University in Toruń, 85-092 Bydgoszcz, Poland; celestyna_mila@cm.umk.pl (C.M.-K.); p.sutkowy@cm.umk.pl (P.S.); roland@cm.umk.pl (R.W.); malgorzata.smogula@gmail.com (M.S.); al1103@cm.umk.pl (A.W.); 2 Department of Health Sciences, Olsztyn University, 10-283 Olsztyn, Poland; boraczynski@osw.edu.pl; 3 Department of Health Sciences, Collegium Medicum, University of Warmia and Mazury, 10-561 Olsztyn, Poland; michal.boraczynski@gmail.com

**Keywords:** cold-water immersion, cytokines, exercise, inflammation, lysosomal enzymes, regeneration method, recovery

## Abstract

Cold-water immersion (CWI) after exercise is a method used by sportsmen to improve recovery. The aim of the study was to assess the effect of a 3 min CWI on the inflammatory state by measuring levels of interleukin 6 (IL-6), interleukin 10 (IL-10), tumor necrosis factor α (TNF-α), and transforming growth factor β1 (TGF-β1), and activities of α1-antitrypsin (AAT) and lysosomal enzymes, including arylsulfatase (ASA), acid phosphatase (AcP), and cathepsin D (CTS D), in the blood of healthy recreational athletes. Male volunteers (*n* = 22, age 25 ± 4.8 yr) performed a 30 min submaximal aerobic exercise, followed by a 20 min rest at room temperature (RT-REST) or a 20 min rest at room temperature with an initial 3 min 8 °C water bath (CWI-REST). Blood samples were taken at baseline, immediately after exercise, and after 20 min of recovery. The IL-6, IL-10, and TNF-α levels and the AAT activity increased significantly immediately after exercise. The IL-6 level was significantly higher after CWI-REST than after RT-REST. No changes in the activities of the lysosomal enzymes were observed. The effect of a 3 min CWI on the level of inflammatory markers during post-exercise recovery was limited. Thus, it might be considered as a widely available method of regeneration for recreational athletes.

## 1. Introduction

Physical exercise is known to enhance and maintain overall health and wellness [1]. However, regular exercise and its effects may induce physical stress. After training, increases in inflammatory marker levels, including interleukin-6 (IL-6), interleukin-10 (IL-10), C-reactive protein (CRP), α1-antitrypsin (AAT), and others, can be observed [2,3]. Moreover, muscular exercise results in an acute increase in the production of reactive oxygen species (ROS), as evidenced by elevated biomarkers of oxidative damage in both the blood and skeletal muscles [4,5]. High levels of ROS lead to membrane lipid peroxidation, protein modifications, and DNA damage [6]. Proinflammatory cytokines, such as tumor necrosis factor α (TNF-α), activate neutrophils and initiate a local inflammatory response. Neutrophils migrate from the blood to damaged myocytes, which is accompanied by the production of ROS [7]. Structural changes in muscle fibers are also accompanied by an increased release of some intracellular enzymes, including lysosomal enzymes participating in intracellular macromolecule digestion [8]. Lysosomal enzymes may be released from cells after physical exercise, affecting the inflammatory response [9]. Taking these processes into consideration, regeneration between training sessions is the key to obtaining high training effectiveness and proper body adaptation to long-term physical effort [6].

Many athletes are interested in regeneration methods based on the exposure of the body to low ambient temperatures, i.e., during a bath in cold water. A rapid increase of body temperature after the end of the low temperature exposure causes increases in the blood flow, leading to better removal of waste metabolites and inflammatory mediators released by damaged tissue [10]. In addition, CWI may reduce muscle tension and fatigue, reduce joint pain, and improve general well-being, thus ensuring better sports performance [11]. Other popular methods of cold therapy include ice packs and whole-body cryotherapy (WBC) sessions [12]. WBC involves exposing the body to extreme low temperature (−100 °C to −160 °C) for 1–3 min. WBC also has a positive effect on the oxidant-antioxidant balance of the athlete’s organism, soothing oxidative stress associated with intense physical effort [8]. However, WBC systems are quite expensive. As CWI is easier to administer for athletes and is a more cost-effective method of recovery with the use of cold than WBC (while providing the same or even greater benefits), it remains the more common method among individuals and teams [13]. There exists a long-standing belief that CWI reduces inflammation in tissues within and around the injured sites in skeletal muscle. Nevertheless, not much is yet known about the effects of cold exposure on pro- and anti-inflammatory cytokines. Moreover, previous research has mainly focused on the effects of CWI on the performance in the professional athlete population, and there is no research on CWI outcomes in the general population and recreational athletes [14]. It is noteworthy that the use of different cooling methods and a wide variety of exercise protocols during experiments resulted in a general dispute as to what types of exercise might benefit from CWI and which method of CWI is the most appropriate [15]. In addition, only a few studies have investigated the effect of post-exercise CWI on inflammatory markers over periods of less than two hours. Thus, establishing the minimum duration of CWI necessary to obtain a beneficial effect on the inflammatory state seems to be an issue of great interest.

Taking into account the unexplained issues of the impact of short CWI on inflammation, the aim of the present study was to determine if a 3 min CWI applied post-exercise is long enough to diminish the level of inflammatory markers in the blood of recreational athletes. For this purpose, concentrations of selected cytokines, including IL-6, IL-10, TNF-α, and transforming growth factor β1 (TGF-β1), as well as activities of AAT and selected lysosomal enzymes, including arylsulfatase (ASA), acid phosphatase (AcP), and cathepsin D (CTS D), were measured in the blood of young healthy recreational athletes who performed a 30 min submaximal aerobic exercise followed by a 20 min rest at room temperature with or without an initial 3 min cold-water bath.

## 2. Materials and Methods

### 2.1. Participants

Twenty-two young healthy recreationally trained male athletes who participated in the summer camp voluntarily agreed to participate in the study. The research included men who had never used cold-water immersion before the study period. The inclusion and exclusion criteria are described in Table 1.

The International Physical Activity Questionnaire (IPAQ) was used to assess the level of physical activity of the subjects during the last 7 days before the study. The IPAQ is expressed in MET × min/week. One MET is equal to 3.5 mL O_2_/min/kg and represents the baseline oxygen consumption [16]. The included participants had a weekly physical activity level ranging between moderate and high. The range of practiced sports or physical workouts included running, strength or cardio workouts in the gym, gymnastics, swimming, tennis, cycling, and football. The category “moderate” means that the participants met at least one of the following criteria: (I) 3 or more days of vigorous activity of at least 20 min per day, (II) 5 or more days of moderate-intensity activity or walking of at least 30 min per day, and (III) 5 or more days of any combination of walking, moderate-intensity, or high-intensity activities, achieving a minimum of at least 600 MET × min per week. The category “high” means that the participants met at least one of the following criteria: (I) vigorous-intensity activity of at least 3 days and accumulating at least 1500 MET × min/week, (II) 7 or more days of any combination of walking, moderate-intensity, or vigorous-intensity activities achieving a minimum of at least 3000 MET × min/week [16]. In the period just before or during the study, the subjects did not change their eating habits as well as the type and intensity of physical activity. People treated for any comorbidities, smoking cigarettes and under therapies that could affect the inflammatory state were excluded from the study. All the participants were informed about the purpose of the research and the potential risks, and they provided informed consent. The subjects were required to complete the baseline examination and two experimental sessions separated by 7 days. The research had the approval of the Bioethics Committee at Collegium Medicum in Bydgoszcz of the Nicolaus Copernicus University in Toruń (KB 278/2016).

### 2.2. Baseline Examination

The anthropometric evaluation and body composition of the participants, including body height (BH, cm), body weight (BM, kg), body mass index (BMI, kg/m^2^), body fat (BF, %), and total body water mass (TBW, kg), were determined using a Tanita bioelectric impedance analyzer—BC 418 MA (Tanita Corporation, Tokyo, Japan). A physical working capacity-170 (PWC170) test was performed to determine the aerobic fitness of the participants [17]. The PWC170 test consisted of two 5 min standard exercise sessions on a bicycle ergometer (Monark Ergomedic 828 E, Vansbro, Sweden). The load for the second exercise test was increased to obtain but not exceed a heart rate (HR) of 170 beats per minute (bpm). The PWC170 index was calculated based on the mean of the HR values recorded at the end of each 5-min exercise period. The HR was measured using a cardiofrequency meter (Polar Electro Oy, Espoo, Finland). The load (power expressed in watts, W) was calculated during exercise at a HR of 170 bpm. The test result was calculated using the following formula: PWC170 = P1 + (P2 − P1)/(170 − HR1) (HR2 − HR1), where P1 means power of the first exercise test, P2—power of the second exercise test, HR1—HR during the first exercise test, HR2—HR during the second exercise test. The value of the PWC170 correlates well with the maximum oxygen consumption (VO_2_ max), which is the primary oxygen function index [18]. The VO_2_ max variables of all participants were calculated according to the Astrand–Ryhming normogram, using the values of PWC170 test [19]. For rating perceived exertion (RPE), the Borg Category Ratio-10 (CR10) scale was used (with a range of values from “0” to “10”). The first rate means “no exertion at all”, whereas the last rate means “extremely strong” effort. There is also an exertion rate over 10, marked as “∗”. It is an exertion that makes the subject “unable to continue” the exercise bout. The RPE scale was used in both study sessions after a 30 min exercise period [20]. All measurements were performed by the same experienced investigator.

### 2.3. Study Design

The research project was divided into two sessions. The participants were assigned to either control condition (Session 1) or cold-water immersion (Session 2) in a counterbalanced crossover order. During Session 1, the volunteers were subjected to a 30 min exercise (70% of the maximum HR, HRmax) on a bicycle ergometer (Monark Ergomedic 828 E), followed by a 20 min rest at room temperature in a sitting position (RT-REST). The HRmax was calculated according to Tanaka’s formula: HRmax = 208 − 0.7 × age [21]. Session 2 consisted of the same physical exercise test (30 min, 70% of the maximum HR, HRmax) and a 20 min recovery, but immediately after exercise, the participants were subjected to a 3-min immersion in a pool of cold water (8 °C) followed by rest at room temperature in a sitting position (CWI-REST). Volunteers were dressed only in swimming suits and they immersed the whole body with the exception of the head and neck. At both sessions, blood samples were taken from the median cubital vein into polypropylene tubes (6 mL) without anticoagulant to obtain serum at baseline (control, BE), immediately after exercise (AE) and after a 20 min recovery (RT-REST or CWI-REST). The samples were transported to a laboratory in a transport refrigerator at 4 °C and then centrifuged (6000× *g* for 10 min at 4 °C). Subsequently, serum was separated and stored at −80 °C for further analysis.

### 2.4. Determination of the Activity of Protease Inhibitor and Lysosomal Enzymes

The activity of AAT was estimated by the Eriksson method [22]. The basis of the assay was a decrease in the enzymatic activity of trypsin due to short incubation with defibrinated blood serum, measured at a wavelength of 410 nm. The activity of AAT was expressed in mg of trypsin-inhibited/mL blood serum. The ASA activity was estimated using the Roy method modified by Błeszyński and Działoszyński [23]. The amount of 4-nitrocatechol (4-NC) released during the enzymatic hydrolysis of 4-p-nitrocatechol sulfate was estimated. The ASA activity was expressed in nmol of 4-NC/mg protein/min. The activity of AcP was measured according to the Bessy method modified by Krawczyński [23]. The amount of p-nitrophenol released during the enzymatic hydrolysis of disodium p-nitrophenylphosphate was estimated and the activity of AcP was expressed in nmol of p-nitrophenol/mg protein/min. The CTS D activity was estimated by Anson’s method [24]. The test sample was incubated with 2% denatured hemoglobin as a substrate at 37 °C and the absorbance at a wavelength of 600 nm was measured and compared to the absorbance of the control. The CTS D activity was expressed in nmol of tyrosine/mg protein/min.

### 2.5. Determination of the Cytokine Concentrations

The concentration of cytokines was estimated using ready-to-use immunoassays: IL-6 (Human IL-6 ELISA KIT, Diaclone SAS, Besancon CEDEX, France), IL-10 (Human IL-10 ELISA KIT, Diaclone SAS, Besancon CEDEX, France), TNF-α (Human TNF-α ELISA KIT, Diaclone SAS, Besancon CEDEX, France), and TGF-β1 (Human TGF-β1 ELISA KIT, Diaclone SAS, Besancon CEDEX, France). The measurements were made according to the manufacturer’s instructions. The cytokine concentrations were expressed in pg/mL or in ng/mL. The sensitivity of the methods, depending on the used calibrates, was 2 pg/mL for IL-6, 4.9 pg/mL for IL-10, 8.6 pg/mL for TGF-β1, and 8 pg/mL for TNF-α.

All the studied parameters were analyzed in duplicate, with sample means taken as the result.

### 2.6. Statistical Analysis

The obtained results underwent univariate analysis of variances (ANOVA) with post hoc statistical analysis (Tukey’s HSD test and Tukey’s assay for different N). To calculate the sample size, the power of 80% and alpha level of 0.05 were used. In the post hoc analysis, all the assumptions of ANOVA (group equality, Levene’s variance homogeneity test, Kolmogorov–Smirnov’s normal distribution test, or Shapiro–Wilk’s test) were considered. The results are presented as an arithmetic mean ± standard deviation (SD). Differences at the level of significance *p* < 0.05 were accepted as statistically significant. The effect size (ES: Cohen’s d) was used to measure the difference between sessions, using the following formula: Cohen’s d = (M2 − M1)/SDpooled, where: SDpooled = √((SD1 + SD2)/2). ES magnitudes were interpreted as follows: <0.2 trivial; 0.2–0.6 small; 0.6–1.2 moderate; 1.2–2.0 large; 2.0–4.0 very large; 4.0 nearly perfect [25].

## 3. Results

### 3.1. Basic Characteristics of the Study Group

The characteristics of the studied group is presented in Table 2. The VO_2_ max value points to the average aerobic fitness of the subjects [18]. Using the Borg Category Ratio-10 scale, the participants evaluated their physical effort during the experiment as “slightly heavy” [20]. No incidents were recorded during exercise sessions or during recovery.

### 3.2. The Concentration of Cytokines

**Figure 1:**
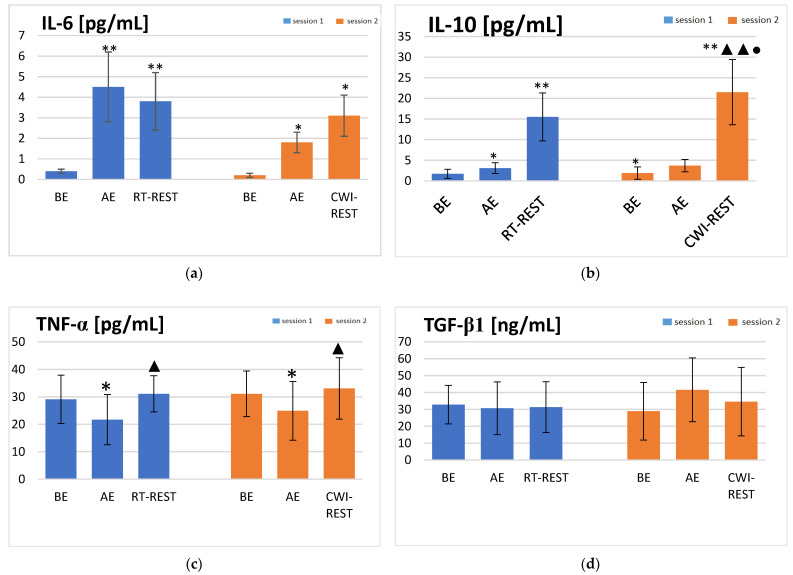
The concentrations of selected cytokines in blood serum of the subjects (young healthy recreational athletes, *n* = 22) during the experiment: (**a**) IL-6 concentration; (**b**) IL-10 concentration; (**c**) TNF-α concentration; (**d**) TGF-β1 concentration. Data are presented as the means ± SD. IL-6—interleukin 6, IL-10—interleukin 10, TNF-α—tumor necrosis factor α, TGF-β1—transforming growth factor β1, BE—before exercise, AF—after exercise, RT-REST—20 min recovery at room temperature, CWI-REST—20 min recovery at room temperature combined with 3 min cold-water immersion, * *p* < 0.05 vs. BE, ** *p* < 0.001 vs. BE, ▲ *p* < 0.05 vs. AE, ▲▲ *p* < 0.001 vs. AE, ● *p* < 0.001 vs. RT-REST.

Statistically significant concentration changes in the investigated cytokines during the session steps were revealed. IL-6 concentration increased more than ten times immediately after exercise (AE) in Session 1 (*p* < 0.001) and was still significantly higher (*p* < 0.001) after 20 min of rest at room temperature (RT-REST) than before exercise (BE) (4.5 ± 1.7, 3.8 ± 1.4, and 0.4 ± 0.1 pg/mL, respectively) (Figure 1a). In Session 2, IL-6 concentration was also nearly ten times higher AE than at baseline (*p* < 0.05), and additionally increased (*p* < 0.05) after 20 min of rest with an initial 3 min cold-water immersion (RT-CWI) (1.8 ± 0.5, 0.2 ± 0.1, and 3.1 ± 1.0 pg/mL, respectively) (Figure 1a). In both sessions, statistically significant increases in IL-10 concentration were found AE (*p* < 0.05) and after 20 min of recovery (*p* < 0.001), when compared to the concentration of this cytokine BE. In Session 1, the concentration of IL-10 was about two-fold higher AE compared to its BE level (*p* < 0.05). Furthermore, the concentration of IL-10 after RT-REST was five-fold higher than AE (*p* < 0.001) and about ten-fold higher than BE (*p* < 0.001) (15.5 ± 5.8, 3.1 ± 1.3, and 1.7 ± 1.1 pg/mL, respectively) (Figure 1b). A similar pattern was observed in Session 2. The concentration of IL-10 was about two-fold higher AE, compared to the BE values (*p* < 0.05). Moreover, the concentration of IL-10 after CWI-REST was five-fold higher than AE (*p* < 0.001) and about eleven-fold higher than BE (*p* < 0.001) (21.5 ± 7.9, 3.7 ± 1.5, and 1.9 ± 1.5 pg/mL, respectively) (Figure 1b). In Session 1, the TNF-α concentration decreased significantly AE (*p* < 0.05). After RT-REST, the concentration of TNF-α statistically increased, as compared to the result obtained AE (*p* < 0.05) (31.1 ± 6.6 and 21.7 ± 9.2 pg/mL, respectively) (Figure 1c). A similar pattern was observed in Session 2. The TNF-α concentration was significantly lower AE, and after CWI-REST, the concentration of this cytokine statistically increased (*p* < 0.05) (24.9 ± 10.7 and 33.1 ± 11.2 pg/mL, respectively) (Figure 1c). No statistically significant changes in TGF-β1 concentration were observed in the course of both sessions (Figure 1d).

The results obtained in both sessions were compared. The concentration of IL-10 was significantly higher (*p* < 0.001) after CWI-REST than after RT-REST (21.5 ± 7.9 and 15.5 ± 5.8 pg/mL, respectively). The ES between session comparisons of the IL-10 concentration was moderate (0.87). The concentration of other cytokines did not differ in a statistically significant manner, comparing RT-REST and CWI-REST. For IL-6, TNF-a, and TGFβ1, the ESs were small (0.57, 0.22, and 0.18, respectively).

### 3.3. The Activity of Protease Inhibitor and Lysosomal Enzymes

**Figure 2:**
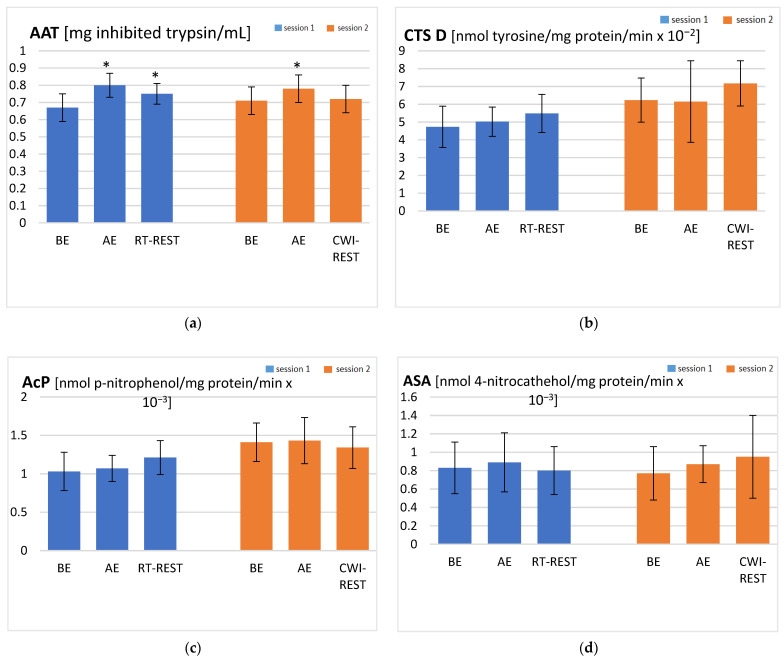
The activities of α1-antytrypsin and lysosomal enzymes in blood serum of the subjects (young healthy recreational athletes, *n* = 22) during the experiment: (**a**) AAT activity; (**b**) CTS D activity; (**c**) AcP activity; (**d**) ASA activity. Data are presented as the means ± SD. AAT—α1-antytrypsin, CTS D—cathepsin D, AcP—acid phosphatase, ASA—arylsulfatase, BE—before exercise, AF—after exercise, RT-REST—20-min recovery at room temperature, CWI-REST—20-min recovery at room temperature combined with 3-min cold-water immersion, * *p* < 0.05 vs. BE.

In Session 1, a statistically significant increase in AAT activity AE was revealed, compared to the activity of this enzyme at baseline (*p* < 0.05). After RT-REST, AAT activity was lower than AE, but it remained higher than BE (*p* < 0.05) (0.75 ± 0.06, 0.80 ± 0.07, and 0.67 ± 0.08 mg inhibited trypsin/mL, respectively) (Figure 2a). In Session 2, the activity of AAT was significantly higher AE than BE (*p* < 0.05) (0.78 ± 0.08 and 0.71 ± 0.08 mg inhibited trypsin/mL, respectively), but decreased to the BE value after CWI-REST (0.72 ± 0.08 mg inhibited trypsin/mL) (Figure 2a). No statistically significant changes in the activities of the lysosomal enzymes were observed in the course of both sessions (Figure 2b–d).

The activity of lysosomal enzymes and AAT did not differ in a statistically significant manner, comparing RT-REST and CWI-REST. For AAT, AcP, and ASA, the ESs were small (0.42, 0.53, and 0.37, respectively) and for CTS D, the ES was large (1.44). The large effect size in the case of CTS D with no statistically significant differences between recovery with and without CWI may indicate that the sample size was not big enough.

## 4. Discussion

The aim of the present study was to determine if a 3 min CWI applied post-exercise is long enough to diminish the level of inflammatory markers in the blood of recreational athletes. Statistically significant increases in IL-6 and IL-10 concentrations were observed in the blood serum of the participants after submaximal exercise. However, a significant post-exercise decrease in TNF-α concentration was observed, followed by a statistically significant increase in the level of this cytokine after RT-REST. Moreover, no statistically significant changes in TGF-β1 concentration were observed. The finding of triggered cytokine production as an effect of physical exercise concurs with many previous studies [26,27,28,29]. Zaldivar et al. [26] observed increased levels of both proinflammatory (IL-1α, IL-2, IL-6, TNF-α, TNF-γ) and anti-inflammatory cytokines (IL-10) after 30 min of physical exercise. Santos et al. [27] reported an increase in IL-10 levels in runners after the marathon, which returned to the pre-workout level after 24 h. Mezil et al. [28] found significant increases in IL-6 and TNF-α concentrations 5 min after low-impact, high-intensity interval exercise, and their return to baseline levels 1 h after exercise. A significant rise in the levels of IL-6 and TNF-α after moderate and strenuous exercise was also revealed by Ambarish et al. [29].

The post-exercise increase in IL-6 levels is well documented in existing literature [30,31,32,33,34,35]. It is worth emphasizing that IL-6 is one of the most rapidly produced myokines as a result of physical exercise, and its levels increase more dramatically than any other cytokine investigated to date [31]. In the present study, the concentration of IL-6 immediately after exercise was as much as 10 times higher than at baseline in either of the studied sessions. What is more, the literature data indicates the stimulating effect of IL-6 on the secretion of IL-10, one of the key anti-inflammatory cytokines [36]. Consistent with these findings, in the present study, an increase in the level of IL-6 was accompanied by an increase in the level of IL-10.

A decrease in TNF-α concentration immediately after exercise, observed in the present study, may indicate that submaximal exercise of the volunteers during the experiment did not cause any significant muscle cell damage. TNF-α concentration is supposed to depend on the secretion of IL-6 as this cytokine inhibits TNF-α expression [36]. However, exercise is likely to suppress TNF-α also via IL-6-independent pathways, as demonstrated by a modest decrease in the level of this cytokine after exercise, found in IL-6 knockout mice [37]. The suppressive effect on TNF-α production may be mediated by β2 adrenergic receptors due to sympatho-adrenergic activation during a single bout of exercise [38]. It may confirm the anti-inflammatory effects of regular exercise. The subjects of the present study were non-professional athletes but they declared a weekly physical activity level ranging from moderate to high. A decrease in the TNFα level as a result of regular moderate exercise was reported by Ambarish et al. [29]. The authors implied that regular moderate exercise optimizes the release of inflammatory cytokines, maintaining them at levels necessary as a buffer to elevate their levels during a sudden burst of exercise.

In the present study, statistically significant post-exercise increases in the AAT activity were observed during both studied sessions. Markovitch et al. [39] demonstrated that the AAT concentration is transiently increased immediately after demanding exercise. An increased activity of AAT after exercise was also reported in the studies of Semple et al. [40] and Schild et al. [41]. The post-exercise increase in the level of IL-6 with a concomitant increase of the AAT activity, found in the present study, may confirm the proposed inflammatory action of IL-6 by stimulation of acute phase protein release [36].

Although many studies have investigated the cytokine response to exercise, only a few of them have validated the anti-inflammatory effects of CWI. A delayed change in the IL-6 level and a decrease in the TNFα level were reported after a 10 min CWI [42] and after a 170 min intermittent CWI [43] in young non-cold-adapted healthy men, but cold exposure was not preceded by exercise. In order to attain the aim of the study, an attempt was made to determine the role of short cold-water immersion, which preceded rest in a sitting position at room temperature, in the modulation of the inflammatory response to submaximal exercise. The beneficial effect of CWI as a post-exercise regeneration method has been presented in many studies [10,11,44]. Several studies report that cold exposure is supposed to aid recovery by attenuating exercise-induced inflammation. However, this mechanism is not well supported in the literature. CWI was found to reduce swelling in athletes subjected to intense exercise [15] and to facilitate the restoration of muscle performance in a stretch–shortening cycle [33]. These effects did not appear to be associated with the modulation of the inflammatory response. Peake et al. [45] suggested that CWI was not more effective than active recovery as far as minimizing inflammation in muscle after exercise is concerned. They observed a post-exercise increase in the intramuscular gene expression of cytokines and neurotrophins. These responses did not differ substantially between cold-water immersion and active recovery. The results obtained in the present study seem to partially confirm the above-mentioned reports. Contrary to our hypothesis, CWI-REST, compared to RT-REST, did not significantly reduce the level of inflammatory markers. Comparing recovery with and without CWI, no statistically significant differences in the level of IL-6, TNF-α, and TGF-β1 were observed. The only statistically significant difference was found in the IL-10 level. The concentration of this cytokine was significantly higher after CWI-REST with moderate ES in comparisons between sessions. IL-10 is considered to be the primary anti-inflammatory agent as it inhibits the production of proinflammatory cytokines by activated monocytes and macrophages [46]. The increased level of IL-10 after CWI-REST, compared to RT-REST, could point to the anti-inflammatory effect of short CWI.

In the present study, the ESs of comparisons between the two recovery methods used were small for most of the measured cytokines. In both sessions, the IL-6 level was statistically higher immediately after exercise. However, a further significant increase in the cytokine concentration was observed only after CWI-REST. This result is in line with the findings of Roberts et al. [32], who revealed an elevated IL-6 level after both CWI and active recovery at 15 and 60 min post-exercise. The up-regulation of IL-6 expression after prolonged cold exposure was also shown by Rhind et al. [47]. Accordingly, CWI following high-intensity sprint exercise did not significantly reduce plasma markers of inflammation in the study of White et al. [33]. The IL-6 level increased significantly immediately after exercise and remained elevated when CWI at both cold (10 °C) and cool (20 °C) temperatures followed the exercise. Moreover, the IL-6 concentration after different CWI modalities was higher than after passive recovery (CON). In the present study, the CWI condition was not associated with reduced cytokine concentrations. However, the up-regulation of the IL-6 level as a consequence of CWI may have a positive aspect. IL-6 acts as a growth factor for skeletal muscle remodeling and regeneration [48]. The increased plasma IL-6 concentration may reflect a sustained release of IL-6 from skeletal muscle in response to CWI-stimulated glycogenolysis [32]. The higher IL-6 level after CWI-REST than after RT-REST may also be due to the modulation of muscle mass as a result of exposure to cold. In contrast, Earp et al. [34] found that the IL-6 level was significantly elevated after 30 min of CON, compared to the CWI session. Differences between the findings described above and in the present study may result from the resistance training status of the participants. In the present study, similar to the research of Peake et al. [49] and Roberts et al. [32], the participants were athletes trained only recreationally, while the participants in the Earp et al. [34] study were resistance trained.

Rhind et al. [47] revealed that, when strenuous exercise preceded exposure to cold, the spontaneous intracellular expression of TNFα was substantially reduced and its serum level was also markedly suppressed in response to cold exposure. The authors suggested that cold-induced changes in the cytokine expression appear to be linked to enhanced catecholamine secretion associated with cold exposure. In non-cold-adapted people, CWI at water temperatures below 15 °C induces a response known as “cold shock”, which can be particularly awkward [42]. This stressful physiological reaction is manifested by increased levels of stress hormones, including cortisol, adrenaline, and noradrenaline [42,43]. CWI may benefit recovery after exercise by inducing vasoconstriction and restricting the infiltration of inflammatory cells into muscle [50,51,52]. According to this mechanism, CWI can reduce clinical signs of inflammation [52]. However, blood plasma levels of cytokines may not be altered at the blood sampling time point. Eimonte et al. [42] observed that although glucocorticoid and catecholamines induce a rapid response, they return to baseline soon after cold exposure. The authors implied that the stress hormones may (re)activate the cytokines gradually, later after CWI. Earp et al. [34] found that the TNFα level after rest with CWI was significantly lower than after CON, but only 15 min post-exercise and not 30 and 60 min after the resistance-exercise bouts. In the present study, the TNF-α level increased significantly to the values observed at baseline after 20 min of both RT-REST and CWI-REST. Both the blood sampling time and the surprisingly lowered TNFα levels after submaximal exercise found in the present study, may explain these discrepancies in the study results. In the study of de Freitas et al. [15], the TNFα levels were comparable between the CWI and placebo groups, but also did not change immediately after exercise, which may suggest that training performed in their study induced no detectable inflammation process in the subjects. It could be assumed that CWI did not reduce the level of TNFα because the exercise load was not sufficient to promote an increase in the level of this inflammatory marker.

In the present study, it was also found that the estimated ES of AAT activity in comparisons between RT-REST and CWI-REST was small. Nevertheless, the AAT activity after CWI-REST was observed to return to the baseline value, while after RT-REST, the AAT activity remained statistically higher than before exercise. It may indicate the beneficial effect of short CWI on the risk of proteolytic tissue damage. The fast response of the AAT activity after different recovery methods, determined in the present study, may confirm its role as an acute phase protein in humans. The primary function of the acute phase response is to protect the organism from further injury and help restore homeostasis [53]. AAT was found to reduce the production of proinflammatory cytokines, inhibit apoptosis, and affect the inhibition of local and systemic inflammatory reactions [54]. It could be inferred that the modulation of cytokine levels might have occurred later than the time points chosen in the present study. Thus, it is highly advisable to extend the blood sampling time point in the experiment protocol to confirm it.

Considering the activities of the lysosomal enzymes, no statistically significant changes were observed in both sessions of the present study. The estimated ESs for ASA and AcP activities were also small. This may indicate that the use of short CWI after physical exercise did not cause lysosomal membrane lability, as the lysosomal enzymes did not leak into the bloodstream. It was proven that lysosomal enzymes are involved in the development of post-exercise muscle fiber damage [7,55]. Lysosomal enzymes in inflammatory infiltrated cells are likely to play a major role in protein catabolism associated with local trauma [56]. The effect of a post-exercise CWI on the lysosomal enzyme activity and the pro- and antioxidant balance in the group of soccer players was determined by Sutkowy et al. [57]. The authors observed that both post-exercise recovery methods, including rest at room temperature and rest at room temperature preceded by CWI, did not significantly affect serum lysosomal enzyme activities, which is in accordance with the results obtained in the present study. No effect on the activity of the lysosomal enzymes was also observed after immersion in a river with a water temperature of 0 °C [58]. As previously mentioned, CWI in water below 15 °C is physiologically stressful and can be particularly hazardous [43]. No changes in the lysosomal enzyme activities in the blood plasma of the participants may suggest that the protocol of using a 3 min CWI in the present study did not cause any disruption of the lysosomal membrane and leakage of the enzymes into the bloodstream.

The limitations of the present study are the small number of the experiment participants and the relatively short duration of cold exposure. Future research should consider extending the cold-water immersion time and collecting blood samples at greater intervals after cold exposure.

## 5. Conclusions

In conclusion, the results of the present study confirm that aerobic exercise induces the inflammatory response of the organism and add to the existing knowledge of the effects of cold-water immersion after exercise. It is worth emphasizing that the effect of CWI on the natural physiological stress and immune responses remains controversial. An effective protocol for using CWI as a method of post-exercise recovery has not yet been well established. The protocols used so far are largely based on individual experience rather than evidence-based research. Moreover, much research in this area is limited to the study of participants who were experienced (adapted) cold-water swimmers, professional athletes who exercised acutely in the cold, or athletes who were previously immersed in cold water as recovery. In the present experiment, the study group consisted of recreational athletes who had never been subjected to cold exposure before, but CWI limited to 3 min seemed harmless to them. The effect of short CWI on the level of inflammatory markers during post-exercise recovery appeared to be limited. Additionally, a slight beneficial effect of a 3 min CWI on the risk of proteolytic tissue damage was observed. Thus, short exposure to cold might be considered as a widely available and cheap method of recovery improvement in recreational athletes. However, the risk of exposure of the human organism to low temperature, in relation to the expected beneficial effects of recovery, as well as the most appropriate CWI protocols must be verified by further studies, in which the time of CWI or the time of blood collection will be extended.

## Figures and Tables

**Table 1:** Eligibility criteria.

Inclusion Criteria
Age 18–30 years Weekly physical activity level ranging between moderate and high Willingness to volunteer to participate in the trial and sign the informed consent form
**Exclusion Criteria**
Cold-water immersion use before the study period Active smoking or illicit drug use Obesity Cardiovascular diseases Pulmonary diseases Energy-restricted diet

**Table 2:** Basic characteristics of the study group (healthy male recreational athletes, *n* = 22).

Parameter	Mean ± S.D.
Age (yr)	25.0 ± 4.8
BH (body height, cm)	179.7 ± 5.0
BM (body mass, kg)	81.4 ± 9.6
BMI (kg/m^2^)	25.3 ± 2.7
BF (body fat, %)	15.6 ± 4.3
TBW (total body water, %)	61.5 ± 3.3
VO_2_ max (maximum oxygen consumption, mL/kg/min) ^1^	40.95 ± 6.6
Borg CR10 (rating of perceived exertion scale) ^1^	4.06 ± 0.8
VO_2_ max (maximum oxygen consumption, mL/kg/min) ^2^	40.67 ± 6.7
Borg CR10 (rating of perceived exertion scale) ^2^	4.08 ± 0.6

^1^ Session 1, ^2^ Session 2.

## Data Availability

The data presented in this study are available on request from the corresponding author. The data are not publicly available due to privacy or ethical restrictions.
